# Safety and Efficacy Profile of Commercial Veterinary Vaccines against Rift Valley Fever: A Review Study

**DOI:** 10.1155/2016/7346294

**Published:** 2016-09-04

**Authors:** Moataz Alhaj

**Affiliations:** Campaign for Control of RVF Disease, Ministry of Agriculture, Gizan City, Saudi Arabia

## Abstract

Rift Valley Fever (RVF) is an infectious illness with serious clinical manifestations and health consequences in humans as well as a wide range of domestic ruminants. This review provides significant information about the prevention options of RVF along with the safety-efficacy profile of commercial vaccines and some of RVF vaccination strategies. Information presented in this paper was obtained through a systematic investigation of published data about RVF vaccines. Like other viral diseases, the prevention of RVF relies heavily on immunization of susceptible herds with safe and cost-effective vaccine that is able to confer long-term protective immunity. Several strains of RVF vaccines have been developed and are available in commercial production including Formalin-Inactivated vaccine, live attenuated Smithburn vaccine, and the most recent Clone13. Although Formalin-Inactivated vaccine and live attenuated Smithburn vaccine are immunogenic and widely used in prevention programs, they proved to be accompanied by significant concerns. Despite Clone13 vaccine being suggested as safe in pregnant ewes and as highly immunogenic along with its potential for differentiating infected from vaccinated animals (DIVA), a recent study raised concerns about the safety of the vaccine during the first trimester of gestation. Accordingly, RVF vaccines that are currently available in the market to a significant extent do not fulfill the requirements of safety, potency, and DIVA. These adverse effects stressed the need for developing new vaccines with an excellent safety profile to bridge the gap in safety and immunity. Bringing RVF vaccine candidates to local markets besides the absence of validated serological test for DIVA remain the major challenges of RVF control.

## 1. Introduction

Rift Valley Fever (RVF) is a life-threatening disease of domestic ruminants and humans, included in OIE list as a notifiable and transmissible disease of serious socioeconomic impacts and public health concerns [1]. The causative agent is Rift Valley Fever virus (RVFV) that belongs to the family Bunyavirridae, genus* Phlebovirus* [2]. It was first reported among livestock in Kenya in 1931; since then it has been reported as occurring in most African countries [3]. The first appearance of RVF virus in new geographical areas outside Africa was reported in Jazan region, southwest Saudi Arabia, in 2000, with 886 confirmed cases involving 124 deaths in humans [4]. The socioeconomic impact of the RVF epidemics has been higher especially to populations that were totally dependent on livestock as source of income. Studies quantifying the socioeconomic impact of RVF outbreaks are lacking.

In Kenya, during 2006/2007 outbreak, the total economic losses from livestock mortality and potential milk production were calculated at over US$9.3 million and US$77,000, respectively. The negative impacts not only affect livestock producers, but also extend to various stakeholders in the marketing chain including livestock traders due to unsold animals during quarantine, slaughterhouses casual laborers, and butchers who were affected by imposition of slaughter bans during outbreaks [5].

As there is no specific treatment for RVF, vaccination of susceptible animals in endemic and high risk areas with safe and cost-effective vaccine during nonepidemic periods remains the only effective method to build sufficient immunity that is able to prevent virus amplification in livestock, break the cycle of transmission, and eliminate the main source of human infection [6]. Although several adverse effects have been associated with vaccination including injection site reactions, systemic and allergic reactions, residual pathogenicity, and genetic recombination [7], the numerous advantages and the benefits derived have promoted the use of vaccines rather than chemotherapy. Apart from the fact that vaccination is the only available method to prevent viral infections in the absence of broad spectrum antiviral, they are mostly environmentally friendly and contribute indirectly to preventing drug resistance and pharmaceutical residues in food [8]. Furthermore, they have significant impacts not only on reducing losses or improving health and production, but also on human health through increasing safe food supplies and preventing zoonotic diseases [9].

A successful vaccination program depends on a proper selection of vaccine, as well as careful handling practices (in accordance with manufacturer's instruction). Vaccine type and timing should be done according to the epidemiological aspect of targeted disease. Generally, live attenuated vaccines are more preferable to inactivated ones, since only a single dose is required to provide a long-term immunity. The live attenuated vaccines are recommended in endemic zones and considered the primary available option for controlling the disease in high risk areas during interepizootic period or at an outbreak early warning phase, while inactivated vaccines are advisable in free low risk zones and free high risk areas [10]. However, during an outbreak time of RVF, vector control, public education, quarantine, and slaughter ban probably are the most effective measures against the disease.

Obviously, the commercial production of RVF vaccines tends to be the biggest challenge, as the cost of sustained vaccination campaigns against RVF is beyond the capacity of most countries suffering regular outbreaks. Additionally, outbreaks of RVF usually occurred at irregular intervals and most commonly following exceptionally heavy rains. These events have led to refusing the annual vaccination during long interepizootic periods which in turn both decreases the demand for vaccines and prevents the manufacturers from maintaining strategic stocks due to limited shelf-life.

It could be argued that reliable information about vaccination in endemic zones is scarce. With the exception of Saudi Arabia, South Africa, and Egypt, all affected countries had not practiced routine vaccination (Table 1). In Egypt control of RVF was based on alternation between live and inactivated vaccines concurrent with periodical vector control. Live vaccine has been used at intermittent periods, before, during, or after outbreaks, in unidentified manner which might be a significant factor in disease persistence and maintaining endemicity of RVF in Egypt [14]. In Jazan region, southwest of Saudi Arabia, has had the hardest hit by the disease in 2000. 65.6% of animal cases occurred in Jazan, 26.9% in Asir, and 7.5% in AlQuenfeda. The infection rate was 23%, 8.7%, and 2% in Jazan, Asir, and AlQuenfeda, respectively [15]. Various control measures since then have been in place including sustaining vaccination campaigns, vector control, and surveillance system. Amazingly, the inactivated vaccine was used during the first three weeks of the outbreak despite the risk of RVFV transmission within and between herds through the reuse of needles during vaccination campaign. The inactivated vaccine subsequently was replaced with live attenuated vaccine (Smithburn strain) which has been used as the gold standard vaccine for several years and seems to play a significant role in control, as long as no clinical disease in humans and animals has been reported yet [16].

Currently, two main types of vaccines with different development techniques are available for immunization against RVF, including live attenuated vaccines and inactivated vaccines [17]. Attenuation of live vaccines was accomplished by in vitro passage through a series of cell cultures so as to produce a version of a virus attenuated to such a level unable to cause disease in animals, together with inducing a rapid onset of long lasting immune response similar to that of natural infection. Inactivation was obtained by growing the virus in culture media before treatment with heat or chemicals such as Formalin to destroy the ability of viruses to replicate [18]. Although inactivated vaccines are biologically safe, are more stable, and have no residual viruses or risk of reversion as attenuated vaccines [19], they are still known to be less protective and to need high antigenic mass and strong adjuvant to stimulate the immune system. Moreover, they continued to be associated with slow onset of immunity, local reactogenicity and residue, risk of incomplete inactivation, and hazards to personnel, as well as not being very efficient without multiple injections [20].

To date, there is no licensed vaccines against RVF available to immunize humans, while various strains for livestock are now licensed and commercially produced including Smithburn vaccine, Formalin-Inactivated vaccine, and Clone13. These vaccines are produced by three different laboratories: Onderstepoort Biological Products limited (OBP) in South Africa, Kenya Veterinary Vaccine Producing Institute (KEVEVAPI), and Egypt's Veterinary Serum and Vaccine Research Institute (EVSVRI) (Table 2).

*Aims of the Study.* This paper specifically aims to

1. summarize commercially available RVF vaccines for veterinary use in Africa and Arabian peninsula,
2. highlight the safety-efficacy profile and drawbacks of these vaccines according to previous safety-efficacy trails.

## 2. Method of Data Collection

A systematic review was conducted by searching Google Scholar (https://scholar.google.com/) and the National Library of Medicine's Medline database through PubMed (http://www.ncbi.nlm.nih.gov/sites/entrez/) up to March 15, 2016. The search terms “RVF” and “vaccine” were combined using the operators “AND” and “OR” to identify the original research articles describing the safety and efficacy profile of commercial veterinary vaccines against RVF. A total of 2619 articles were identified by searching Google Scholar and PubMed (2510, 109), respectively. The identified studies were screened on the basis of original research and its relevance to the aim of this review; in addition the full article should be published in English-language. Studies that did not meet inclusion criteria were excluded.

Of 2619 screened reports, 31 articles were finally selected on the basis of inclusion criteria to describe Smithburn vaccine (12 articles), inactivated vaccine (9 articles), and Clone13 (10 articles). Additional studies were obtained through citation tracking of review and original articles.

### 2.1. Smithburn Vaccine

Smithburn Vaccine Strain was derived from the virulent Entebbe strain, isolated from mosquitoes in Uganda and developed by serial passages in mouse brains to be able to induce immunity in ewes and their offspring after subcutaneous inoculation [21], currently produced in OBP and KEVEVAPI in freeze-dried form. The recommended dose is 1 mL of the reconstituted vaccine administered via subcutaneous route for the immunization of sheep and goats for OBP vaccine whereas cattle received 2 mL of RIFTVAX TM vaccine compared with 1 mL of Rift Valley Fever (Live) produced at OBP. According to manufacturer's instructions, the vaccine can cause abortion or fetal malformation in a small percentage of animals, particularly sheep, as well as a slight febrile reaction that may occur on the second to fourth day following inoculation. Accordingly, the use should be restricted to nonpregnant animals above six months of age before or at the mating season so as to ensure maternal antibodies and to avoid abortion as well [22]. Despite these adverse outcomes, it has been widely used for many years as the major prevention measure as a cost-effective vaccine in most endemic zones, since the first introduction of the virus [23]. Likewise, in Jazan region, Saudi Arabia, it has been used as the gold standard vaccine for several years as a prevention measure, since 2000 outbreak. It has also been proved through serological surveys to be effective and highly beneficial in controlling infections, as no notable clinical signs in animals have been reported yet [24]. Published efficacy studies conducted in the same region in sheep and goats reported that the vaccine was highly immunogenic and able to induce long lasting antibodies, irrespective of variations among vaccine batches. The level of herd immunity induced by Smithburn Strain Vaccine significantly declined with elapse of years. The percentage of IgG positive animals declined from 95% to 66.7% after one year, and it would decline to zero after six years and eleven months [25]. This decline could be as the consequence of low sensitivity of ELISA test over time. The IgG sandwich ELISA was more sensitive and highly accurate in yearly diagnosis of infection or vaccination with RVF [26]. On the contrary, some safety and potency concerns associated with Smithburn vaccine. The vaccine neither was able to produce proper protective antibodies in all animal species particularly cows, nor was safe in immunocompromised animals and pregnant ones during gestation period leading to high rate of abortion [27]. Larger efficacy and safety study conducted to investigate antibody response to Smithburn vaccine in cattle reported that twenty-eight cows out of 120 pregnant cows and buffalos aborted within three days after vaccination. Moreover, the isolation of the virus from aborted fetus has proved in utero transmission of the vaccine virus [27]. Furthermore, the vaccine virus not only caused abortion and death of fetus at parturition, but also caused harmful changes in internal organs and propagated inside hepatic cells in a manner similar to natural infection [28].

### 2.2. Formalin-Inactivated Vaccine

The lyophilized vaccine containing 2% Human Serum Albumin was first prepared in African green Monkeys Kidney cell and proved to be safe, immunogenic, and highly resistant to thermal deterioration [29]. Commercially produced from OBP and EVSVRI, the virus strain was adapted for growth in baby hamster Kidney (BHK-21) cell, with aluminum hydroxide gel adjuvant for immunization of cattle, sheep, and goats, irrespective of the age and stage of pregnancy [12]. A safe version of inactivated vaccines with minor side effects named TSI-GSD 200 was developed in USA by using a new master seed of the Entebbe strain. The vaccine is neither licensed for use in human nor commercially available but has been used to protect personnel who either work in laboratories with RVFV or would be exposed to RVF infection, after receiving three doses on days 0, 7, and 28, to provide good long immunity with neutralizing antibody titers (1 : 140) [30]. The safety and efficacy profile of inactivated vaccines have been further investigated in several trials. The immunization of susceptible cattle, sheep, and goats with inactivated vaccine would induce higher neutralizing antibodies persisting for 9 month in cattle with evidence of protections against RVFV in pregnant ewes [31]. A comparative study conducted to assess the response in cattle to live and inactivated RVF vaccines revealed that a booster dose of inactivated vaccine after 3 months of the first vaccination was safe and able to evoke a good response sufficient to protect cattle against RVF for at least 1 year [32]. Further studies were conducted to evaluate the inactivated OBP vaccine as it is extremely difficult to maintain low temperatures during vaccine transportation. The vaccine was stored in different temperatures (4°C, 25°C) with alternation between 4°C and 25°C for a week. It was suggested that the vaccine was stable, well tolerated with mild or limited adverse reactions, and not adversely affected by variation in temperature during transportation and that induced long-term neutralizing antibodies may persist for 21 months after booster dose at any age and any stage of pregnancy [33, 34].

### 2.3. Clone13 Vaccine

Although, Formalin-Inactivated vaccine and live attenuated Smithburn vaccine are widely used in control, both of them were accompanied by significant concerns. The first one requires multiple doses for protection, and the second has a risk of causing abortion and fetal malformation in pregnant animals [35]. Drawbacks of these vaccines stressed the need for alternative vaccines in terms of safety and efficiency. Consequently, a massive progress and several initiatives have been done for the evolution of modern vaccines. Recent studies have shown that RVF virus vaccines containing deletions of the NSs and NSm genes are highly attenuated, confer protective immunity with no detectable viremia, and could be useful in control of RVF virus in endemic regions, as well as allowing for DIVA [36]. The commercial OBP vaccine named (RVF Clone13) has recently been registered, marketed in a form of freeze-dried live attenuated virus (Clone13 strain), and extensively used in South Africa [37]. Clone13 is a naturally attenuated isolate of RVF virus with a large deletion in the S segment. It was cloned by plaque purification of nonfatal human case isolate (74HB59 strain), obtained during 1974 RVF outbreak in Central African Republic, and proved to be highly immunogenic leading to long-term immunity as well [38]. Published efficacy and safety studies of Clone13 vaccine have shown that the vaccine protects animals properly without inducing undesirable clinical signs, such as abortion in pregnant ewes, pyrexia, or fetal malformation in their offspring [39]. Recent efficacy and safety studies conducted in sheep and goats in Senegal stipulated that the vaccine was safe at stages of pregnancy and did not induce adverse effects. Additionally, more than 70% of the vaccinated sheep and goats showed that long-term seroconversion persisted for 1 year after vaccination [40]. However, some safety studies raised concerns about the possibility of genetic reassortant between S segment in Clone13 vaccine and virulent strains in field [41]. Furthermore, in a more recent study, it was reported that the vaccine virus is able to cross the ovine placental barrier and spread to the fetus resulting in malformations and stillbirths [42]. Remarkably, the vaccine has the potential to be used as DIVA vaccine for RVF, but the accompanying diagnostic tests are not yet commercially available [43].

Although the currently available commercial vaccines have made great contributions to RVF control over the past 80 years, they are associated with some safety and efficacy concerns, including, but not limited to, risk of abortion, pyrexia, fetal malformation, teratogenic effects, viraemia, risk of reassortment, short shelf-life, revaccination, and risk of incomplete inactivation in killed vaccines (Table 3). The gap in the safety and immunity explains the need for new promising candidates currently under development, such as subunit vaccines, virus vector, and replicons [44, 45]. The most prominent among these candidates is a recombinant* Capripoxvirus* (CPV) vaccine which was developed to protect against RVFV as well as against sheep poxvirus infection. Promising results have been reported in Preclinical Stage trials including safety in pregnant ewes and offspring, stability of the vaccine, and its potential for DIVA [46].

## 3. Conclusion

To sum up, the study has come out with some important results which can be summarized as follows.

First, commercial vaccines currently available in the market are lacking safety and DIVA.

Second, live attenuated Smithburn was reported to cause abortion and fetal malformation in pregnant ewes.

Third, Formalin-Inactivated vaccine requires multiple doses or annual revaccination to provide protection which renders the vaccine not recommended in endemic zones.

Fourth, the safety of Clone13 during the first trimester of gestation remains controversial as it has been reported that the vaccine causes malformations and stillbirths.

Fifth, drawbacks of currently available vaccines stress the need for developing and bringing vaccine candidates to markets in near future to fill the gap in safety and immunogenicity.

Finally, validated serological test for DIVA should be considered in future researches.

## Figures and Tables

**Table 1:** Vaccination program in Africa and Arabian Peninsula [11].

Country	Type of vaccine	Vaccine schedule	Historical outbreaks
Saudi Arabia	Live attenuated (Smithburn strain)	Annual vaccination	2000
South Africa	Live attenuated (Clone13)	Annual vaccination (high risk zones)	1950, 1974, 1981, 1996, 1999, 2010
Egypt	Inactivated vaccine	Biannual vaccination	1977, 1993, 1996, 2003
Kenya and Tanzania	Live attenuated (Smithburn strain)	At outbreak warning	1931, 1936, 1968, 1978, 1997, 1951, 2006

**Table 2:** Commercially available vaccines against RVF [12].

Commercial vaccine name	Vaccine type	Form	Company	Adjuvant	Packing	Instructed dose	Use in pregnant animals
Rift Valley Fever (inactivated)	Inactivated	Liquid	*Onderstepoort Biological Products*	Aluminum hydroxide	Available in bottles of 100 mL	Sheep and goats 1 mL s/c Cattle 2 mL s/c	Yes
Rift Valley Fever (live)	Live attenuated	Freeze-dried	*Onderstepoort Biological Products*	No	Available in bottles of 100 doses	Cattle, sheep, and goats 1 mL s/c	No
RVF Clone13	Live attenuated	Freeze-dried	*Onderstepoort Biological Products*	No	Available in bottles of 100 doses	Cattle, sheep, and goats 1 mL s/c	Yes
Rift Valley inactivated vaccine	Inactivated (Zagazig H501 strain)	Liquid	*Veterinary Serum and Vaccine Research Institute*	Aluminum hydroxide	Available in bottles of 100 doses	Sheep and goats 1 mL s/c Cattle 2 mL s/c	Yes
RIFTVAX TM	Live attenuated Smithburn strain	Freeze-dried	*Kenya Veterinary Vaccine Producing Institute*	No	Available in bottles of 100 doses	Sheep and goats 1 mL s/c Cattle 2 mL s/c	No

**Table 3:** Safety and efficacy profile of commercial vaccines against RFV [13].

Commercial vaccine name	Safety profile	Persistence duration of antibodies	Cost
Rift Valley Fever (live)	Cause abortion in pregnant ewes Cause teratogenic effects Cause significant level of viraemia Risk of reversion to virulence	Long-term immunity Single dose	Low price

RVF Clone13	Safe in pregnancy Very low viraemia Risk of genetic reassortment Restricted to endemic zones	Short shelf-life Single dose Lon-term immunity	Low price

Rift Valley Fever (inactivated)	Safe in pregnancy Can be used during outbreaks Can be used in low risk zones	Booster dose is required Annual revaccination Not practical in routine vaccination	High cost
